# Electronic health records in nursing from 2000 to 2020: A bibliometric analysis

**DOI:** 10.3389/fpubh.2023.1049411

**Published:** 2023-02-09

**Authors:** Ze Luan, Zhiru Zhang, Yanan Gao, Shiyuan Du, Nan Wu, Yulu Chen, Xin Peng

**Affiliations:** ^1^School of Nursing, Jilin University, Changchun, China; ^2^The First Hospital of Jilin University, Bethune First Hospital of Jilin University, Changchun, China

**Keywords:** electronic health records, bibliometric analysis, nursing, data visualization, CiteSpace

## Abstract

**Background:**

Electronic health records (EHR) is the longitudinal data generated by patients in medical institutions and recorded by electronic medical information systems in the form of digital, which is also the most widespread application of big data in medicine. The purpose of this study was to explore the application of electronic health records in the field of nursing and determine the current research status and hotspots.

**Methods:**

A bibliometric analysis of electronic health records in nursing was undertaken from 2000 to 2020. The literature comes from Web of Science Core Collection database. We used CiteSpace (version 5.7 R5; Drexel University), which is a Java-based software that especially visualized collaborative networks and research topics.

**Results:**

A total of 2616 publications were included in the study. We found that publications increased year by year. The *Journal of American Medical Informatics Association* (*n* = 921) is the most cited. The United States (*n* = 1,738) has the most publications in this field. University Penn (*n* = 63) is the institution with the most publications. There is no influential cooperation network among the authors, of which Bates, David W (*n* = 12) have the largest number of publications. The relevant publications also focus on the fields of health care science and services, and medical informatics. In keywords, EHR, long-term care, mobile application, inpatient falls, and advance care planning has been researching hotspots in recent years.

**Conclusion:**

With the popularization of information systems, the publications of EHR in the nursing field have increased year by year. This study provides the basic structure, potential cooperation, and research trends of EHR in the field of nursing from 2000 to 2020, and provides a reference for nurses to effectively use EHR to help clinical work or scientific researchers explore the potential significances of EHR.

## 1. Introduction

With the popularization of medical information systems, the inadequacy of paper records has been exposed, Electronic Health Records (EHR), as a carrier of clinical information, have played an important role in medical information systems (1). Although EHR has been standardized and defined, they lack consistency due to different countries and organizations (2). Adler-Milstein, Holmgren, etc. show that demographics, doctor notes, nursing assessment, patient problem lists, patient medication lists, discharge summary, radiology reports, laboratory reports, diagnostic results, and order entry are hospitals with a basic EHR system that should include (3). EHR can make it easier for researchers to access and aggregate clinical data. The application of EHR in clinical nursing can reduce medication errors, improve patient medication compliance, and reduce nurses' work burden (2, 4). In addition, researchers are paying more and more attention to the potential of EHR. With the development of artificial intelligence, researchers are using machine learning algorithms to analyze EHR to predict disease progression, complications and mortality, and for early diagnosis, self-care, preventive care, clinical decision support and so on (5, 6). During the coronavirus disease 2019 (COVID-19) pandemic, Tortolero et al. leveraged an EHR system to share data to coordinate patient care (7), and Satterfield et al. used it for disease surveillance and contact tracing (8). The COVID-19 pandemic has more clearly demonstrated the need for global data sharing, even though the sharing of disparate COVID-19 data (e.g., genetic sequences, epidemiology, etc.) has been allowed, but beyond that the accurate collection and reuse of EHRs still difficult (9). The use of EHRs has increased rapidly over the past few decades, and there is a growing need for nursing to gain new insights from data to provide evidence-based care. However, the research topics and trends of EHR in the field of nursing are unclear. Therefore, as an important member of the medical team, nurses are also the input and executor of EHR, and determining the future development direction of EHR in nursing is an important task from which they can benefit (10).

Bibliometrics provides a tool for analyzing a large number of publications to more conveniently analyze the development trend, including countries, institutions, authors, citations and so on. There are many studies using bibliometrics to analyze research trends, especially during the wide epidemic period of COVID-19. Ahmad used bibliometrics to analyze the research trends of SARS-CoV-2 and COVID-19 in the previous 2 years (11), and Zhang et al. analyzed the global research trend of COVID-19 nursing (12). In addition, bibliometrics is also used in vaccine development (13), clinical intervention (such as diabetes) (14) and any other fields. However, at present, there is no research on EHR in the nursing field in the way of bibliometrics. Therefore, to fill this gap, the purpose of this study was to use a bibliometrics approach to analyze the literature related to EHR in the field of nursing from 2000 to 2020 to identify research trends in this area in order to gain more insight.

## 2. Methods

### 2.1. Bibliographic search

The data used in the current study was downloaded from the Web of Science Core Collection (WoSCC) database and was published between 2000 and 2020. We use advanced search methods, search #1 AND #2, where #1 representing EHR, #2 denoting nursing. In detail, #1 is TS= (“Computerized Medical Records” OR “electronic medical record” OR “EMR” OR “electronic patient record” OR “electronic medical records” OR “electronic health record” OR “EHR” OR “electronic health records” OR “EHRs” OR “EMRs” OR “electronic patient records”). #2 is TS=(nurs^*^). A total of 3,065 literature data were collected.

### 2.2. Data processing

**Figure 1:**
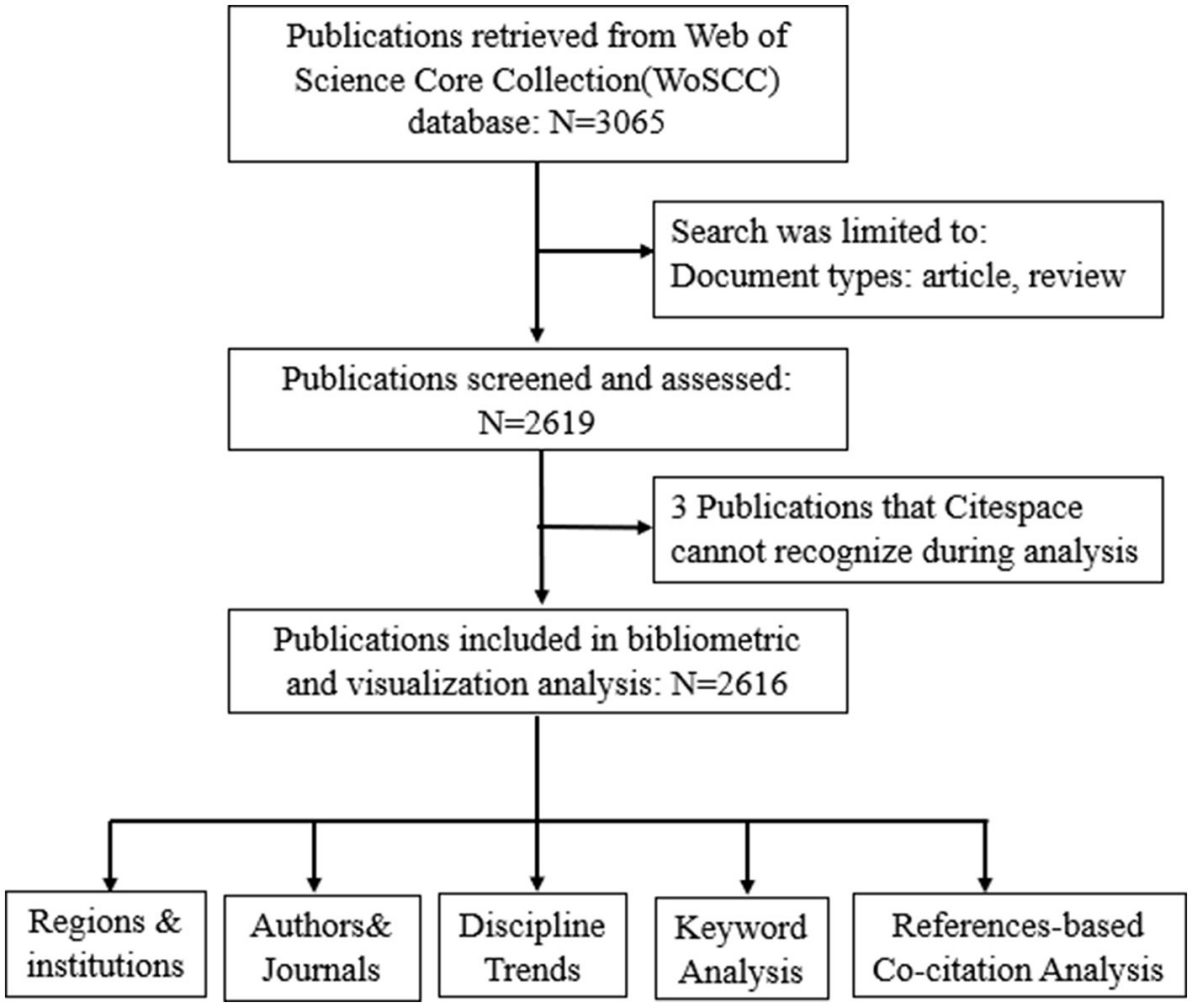
Articles selection flow chart.

The search time was January 18, 2021, and a total of 3065 records were retrieved from the WoSCC. After screening the publication type as review and article, 2619 publications were derived from WoSCC, and the contents of the publications were full records and cited references. The deduplication function of CiteSpace was used to output 2,616 unique records (Figure 1).

### 2.3. Visualization analyses

For the analysis, we used CiteSpace (version 5.7 R5; Drexel University) developed by Dr. Chen based on Java software (15). This is software that allows analysis of collaboration networks in specific fields and visualization of research topics and presents the structure, law and distribution of scientific knowledge through visual means. It is easy to learn and the latest scientific trends and mapping technologies are mostly from CiteSpace. In our opinion, it has two advantages: First, CiteSpace can conduct co-citation analysis in addition to basic analysis and clustering. Second, it is aesthetically scientific from a visual perspective, and the timeline is a feature of it.

The time slices are set to January 2000 to December 2020, and the year of each slice is set to 1 year. Set up different nodes according to the analysis content. For example, select the “author” node when analyzing the author cooperation network, select the “keyword” node when analyzing the keyword co-occurrence network, and select references to describe the collaborative network and cluster analysis. Then, in different types of visual calculation results, a node can represent the above-mentioned different node meanings. Any node with a centrality value ≥0.1 is considered significant. Purple nodes represent milestones for that node. The meaning of the red circle is that it is heavily cited in the short term. The link between two nodes is an edge, which represents a cooperative network. Understanding these will help us analyze the results to determine important directions and hotspots in the retrieval field (15, 16).

### 2.4. Ethics statement

As this study was conducted without any human or animal subjects, ethical consideration was not required.

## 3. Results

### 3.1. Annual trends in publications and citations

**Figure 2:**
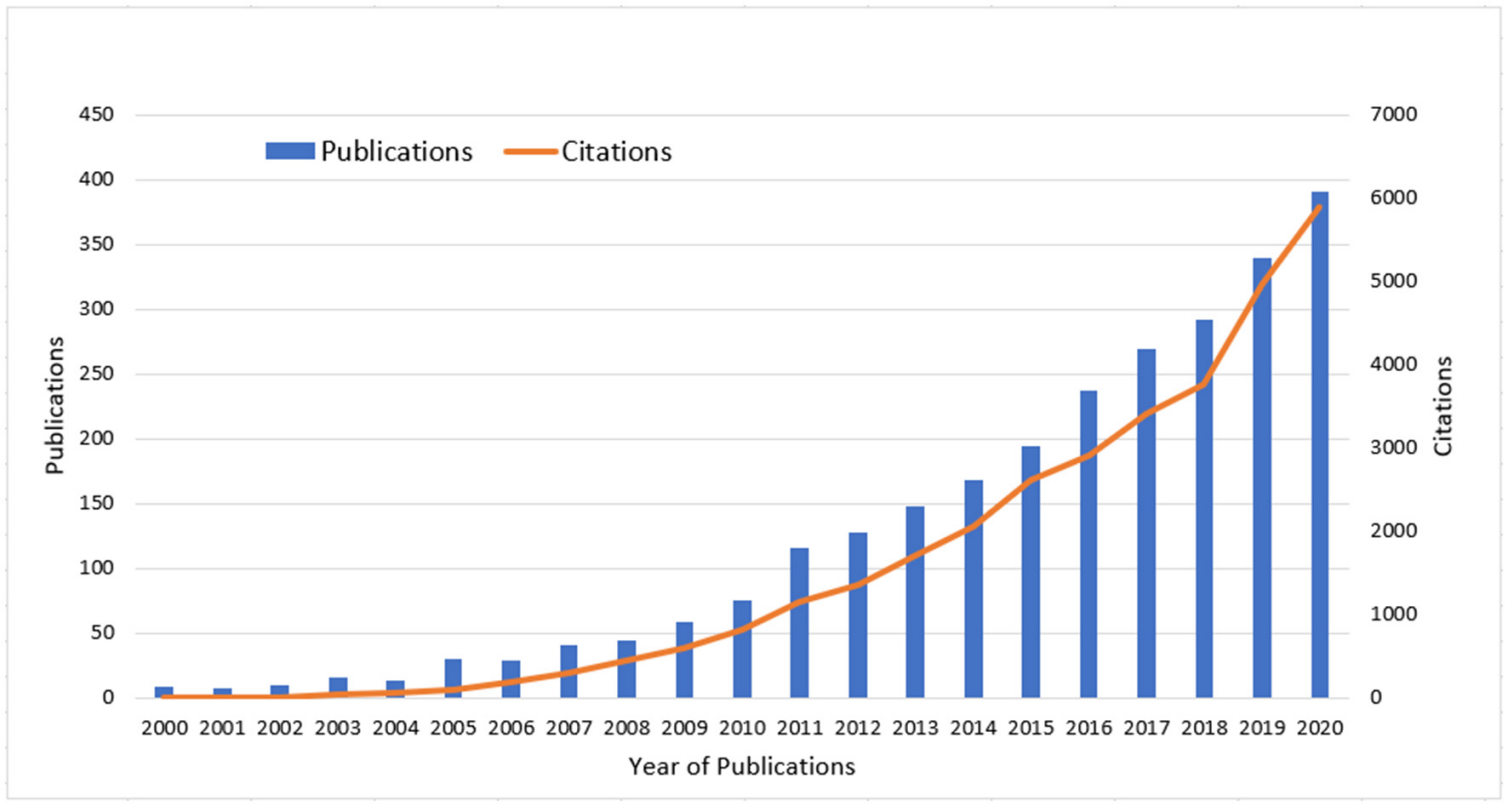
Publication and citation trends of papers in WoSCC database.

From 2000 to 2020, WoSCC database retrieved a total of 2,616 papers, with a total h-index of 70 and an average of 12.42 citations. The number of co-cited papers is on the rise (Figure 2). Publications were published in 237 journals, and 688 authors from 293 institutions in 44 countries were included these publications received 32479 citations. The most published and cited years are 2020, 388, and 5,890 respectively (Table 1).

**Table 1:** Year of publications and citations.

**Publications year**	**Number of published papers**	**Percentage**	**Citations**	**Percentage**
2000	9	0.34%	0	0.00%
2001	8	0.31%	11	0.03%
2002	10	0.38%	17	0.05%
2003	16	0.61%	41	0.13%
2004	14	0.54%	72	0.22%
2005	30	1.15%	107	0.33%
2006	29	1.11%	185	0.57%
2007	41	1.57%	303	0.93%
2008	45	1.72%	451	1.39%
2009	59	2.26%	608	1.87%
2010	76	2.91%	830	2.56%
2011	116	4.43%	1164	3.58%
2012	128	4.89%	1362	4.19%
2013	148	5.66%	1708	5.26%
2014	168	6.42%	2059	6.34%
2015	194	7.42%	2617	8.06%
2016	237	9.06%	2909	8.96%
2017	269	10.28%	3408	10.49%
2018	292	11.16%	3765	11.59%
2019	339	12.96%	4972	15.31%
2020	388	14.83%	5890	18.13%

### 3.2. Top 10 leading countries, authors and institutions

**Figure 3:**
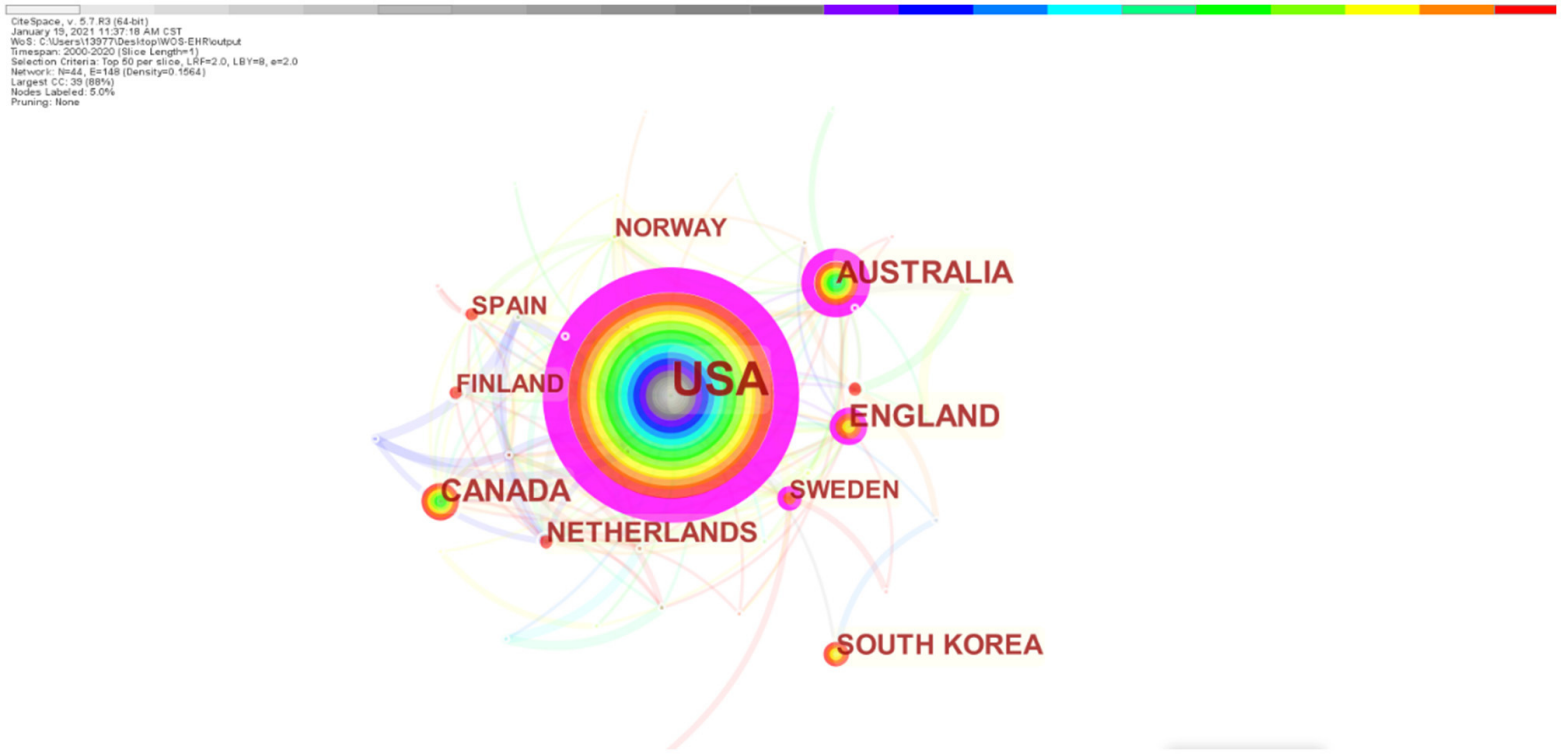
Regional collaborative networks.

From a regional perspective, as shown in Figure 3 and Table 2, the 10 countries with the highest number of publications are the United States, Australia, Canada, the United Kingdom, the Netherlands, South Korea, Sweden, Spain, Finland, and Norway, represented by the node size. Among them, the four countries of the United States, Australia, Sweden, and the United Kingdom have a centrality >0.1, which is shown in purple in the figure, which means that they have an important influence on pioneering research in the EHR global nursing field cooperation. The color of the circle corresponds to the color of the ribbon above the picture, from 2000 to 2020.

**Table 2:** Top 10 leading countries, institutions and authors of EHR research in the field of nursing.

**Top**	**Frequent**	**Centrality**	**Countries**	**Frequent**	**Centrality**	**Institutions**	**Frequent**	**Centrality**	**Authors**
1	1738	0.74	The United States	63	0.12	University Penn	12	0	Bates, David W
2	127	0.26	Australia	55	0.1	University Minnesota	12	0	Monsen, Karen A
3	106	0.02	Canada	53	0.05	University Colorado	10	0	Hypponen, Hannele
4	93	0.12	England	51	0.18	University Michigan	10	0	Keenan, Gail M
5	74	0.06	Netherlands	50	0.11	Duke University	10	0	Stifter, Janet
6	69	0	South Korea	49	0.18	Harvard Medical School	10	0	Wilkie, Diana J
7	52	0.14	Sweden	47	0.08	Brigham and Women's Hospital	9	0	Bowles, Kathryn H
8	49	0.03	Spain	40	0.1	Columbia University	9	0	Saranto, Kaija
9	48	0	Finland	40	0.09	Ohio State University	9	0	Yao, Yingwei
10	44	0.02	Norway	39	0.05	University Wisconsin	8	0	Bakken, Suzanne

From the perspective of the author, there are no authors with centrality >0.1, so we only provide the top 10 authors in Table 2 by the number of publications.

**Figure 4:**
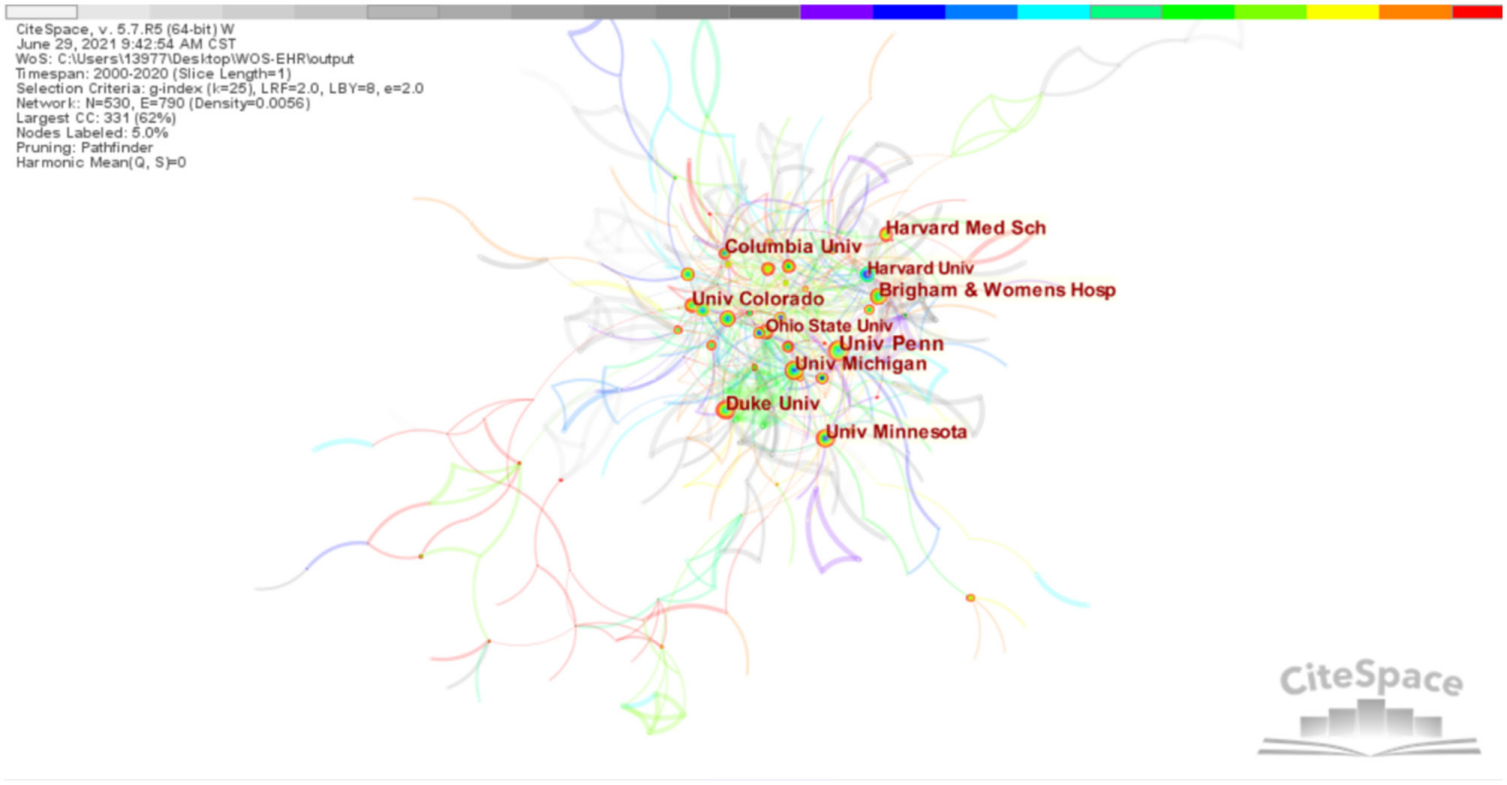
Institutions collaborative networks.

From the perspective of the author's institution, there are a total of six universities with centrality ≥0.1 (Figure 4). Additionally, Figure 4 shows collaborations among the top ten institutions, identified by different colors. They are University Michigan, University Minnesota, Harvard Medical School, University Penn, Duke University, University Minnesota, Columbia University. The details of the top 10 countries and institutions are shown in Table 2. All institutions are in the United States.

### 3.3. Top 10 leading cited journals

**Figure 5:**
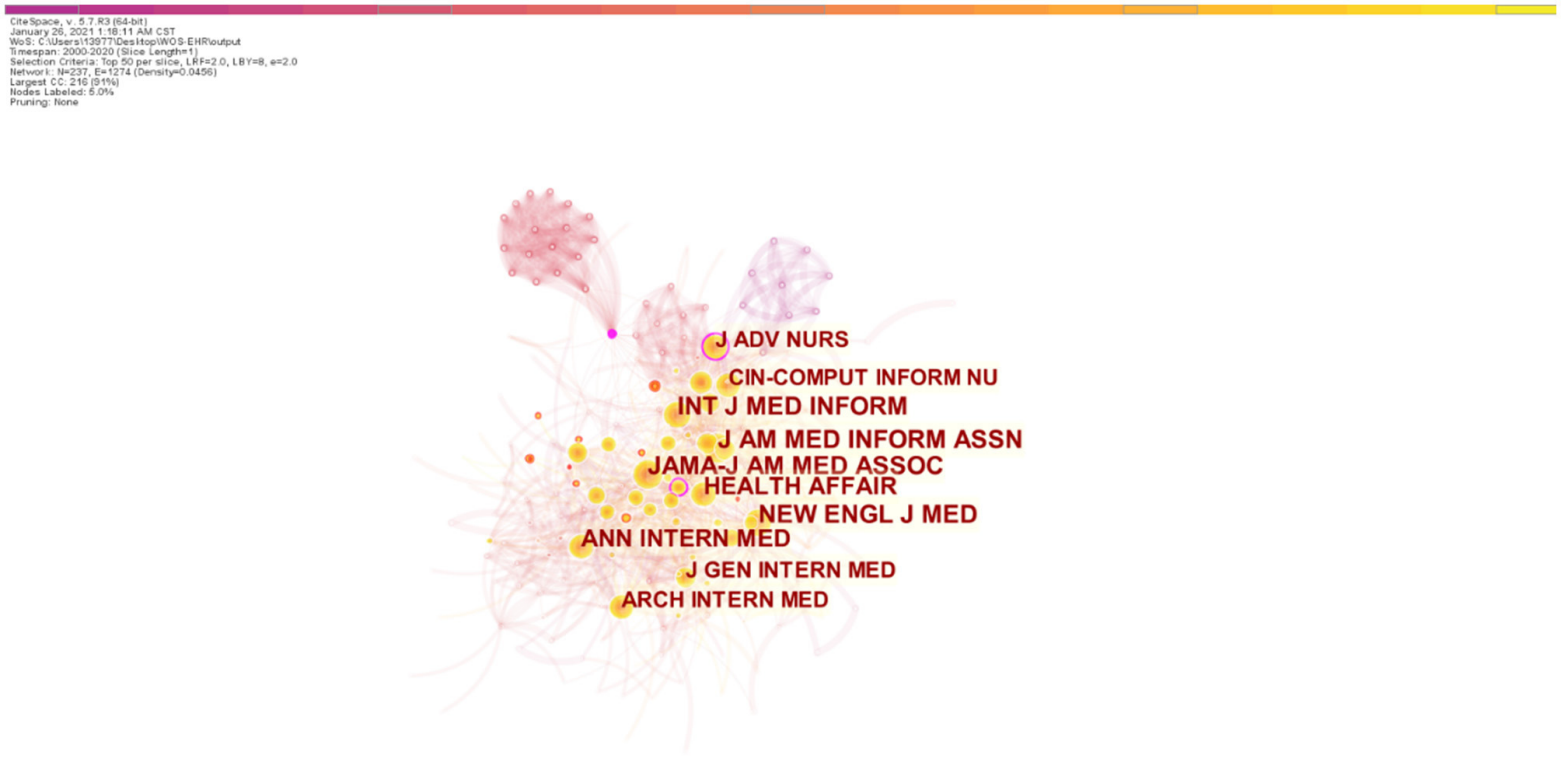
Journals collaborations of EHR in the nursing field.

Figure 5 below shows the top 10 cited journals, in descending order: *Journal of American Medical Informatics Association* (921), *JAMA-Journal of the American Medical Association* (864), *New England Journal of Medicine* (748), *International Journal of Medical Informatics* (666), *Annals of Internal Medicine* (551), *Health Affairs* (496), *Journal of advanced nursing* (444), *CIN-Computers Informatics Nursing* (439), *Journal of General Internal Medicine* (436) and *Archives of Internal Medicine* (413). Besides, four nodes whose centrality is >0.1 are purple, and the corresponding journals are *Research in Nursing & Health, Journal of Advanced Nursing, AMIA Annual Symposium Proceedings, and Annals of Internal Medicine*.

### 3.4. Discipline-specific trends

**Figure 6:**
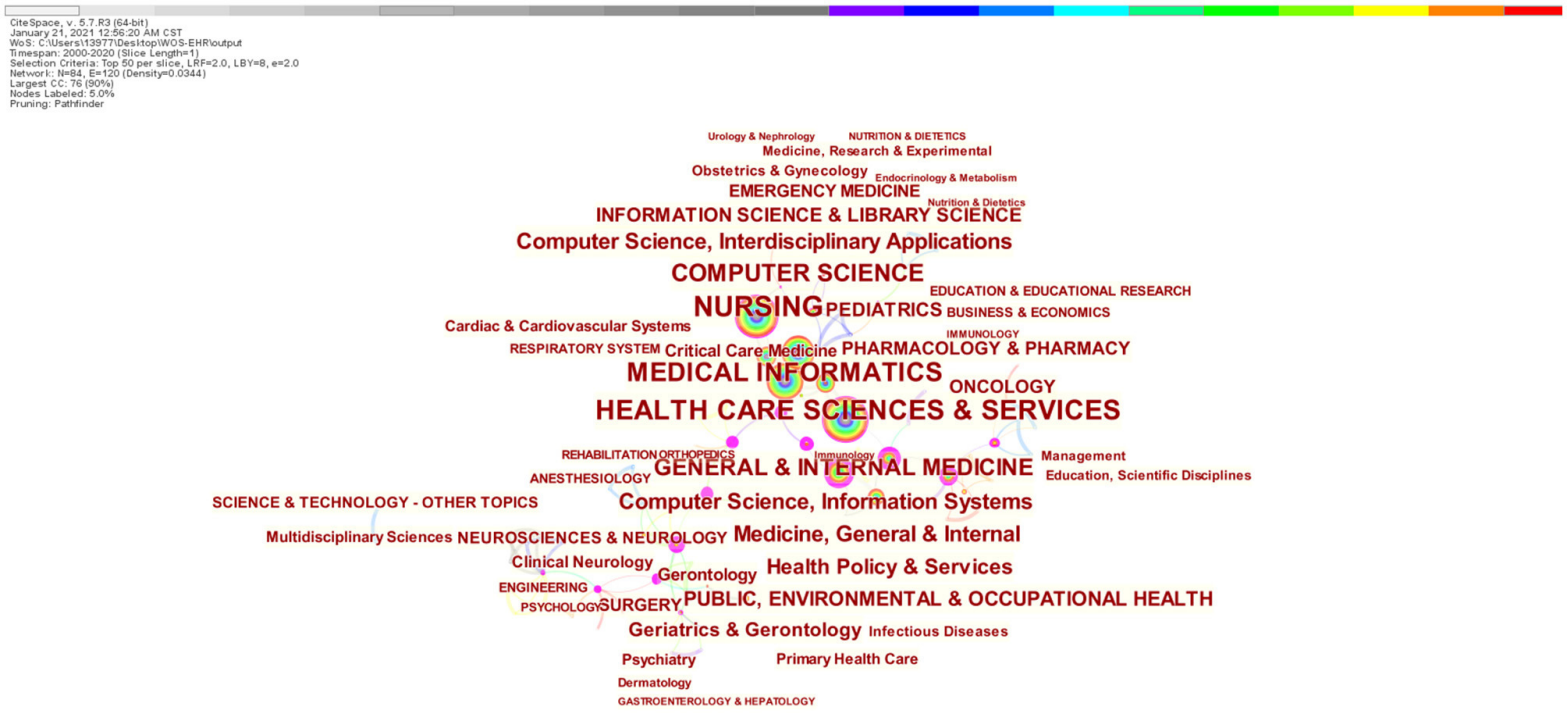
Categories collaborative networks.

The analysis is shown in Figure 6 that health care science and services (*n* = 672), medical informatics (*n* = 606), and computer science (*n* = 402) reported the highest number of publications. The font size and thickness represent the frequency, the larger the word, the more frequency. The following 19 disciplines have a centrality value >0.1, resulting in the collaborative impact of important innovations, in descending order: respiratory system, neurosciences and neurology, cardiac and cardiovascular systems, neurosciences, health policy and services, critical care medicine, general and internal medicine, psychiatry, public environmental and occupational health, psychology, allergy, engineering, oncology, pediatrics, surgery, health care sciences and services, obstetrics and gynecology, medicine, research and experimental and nursing (Figure 6).

### 3.5. Keyword analysis

**Figure 7:**
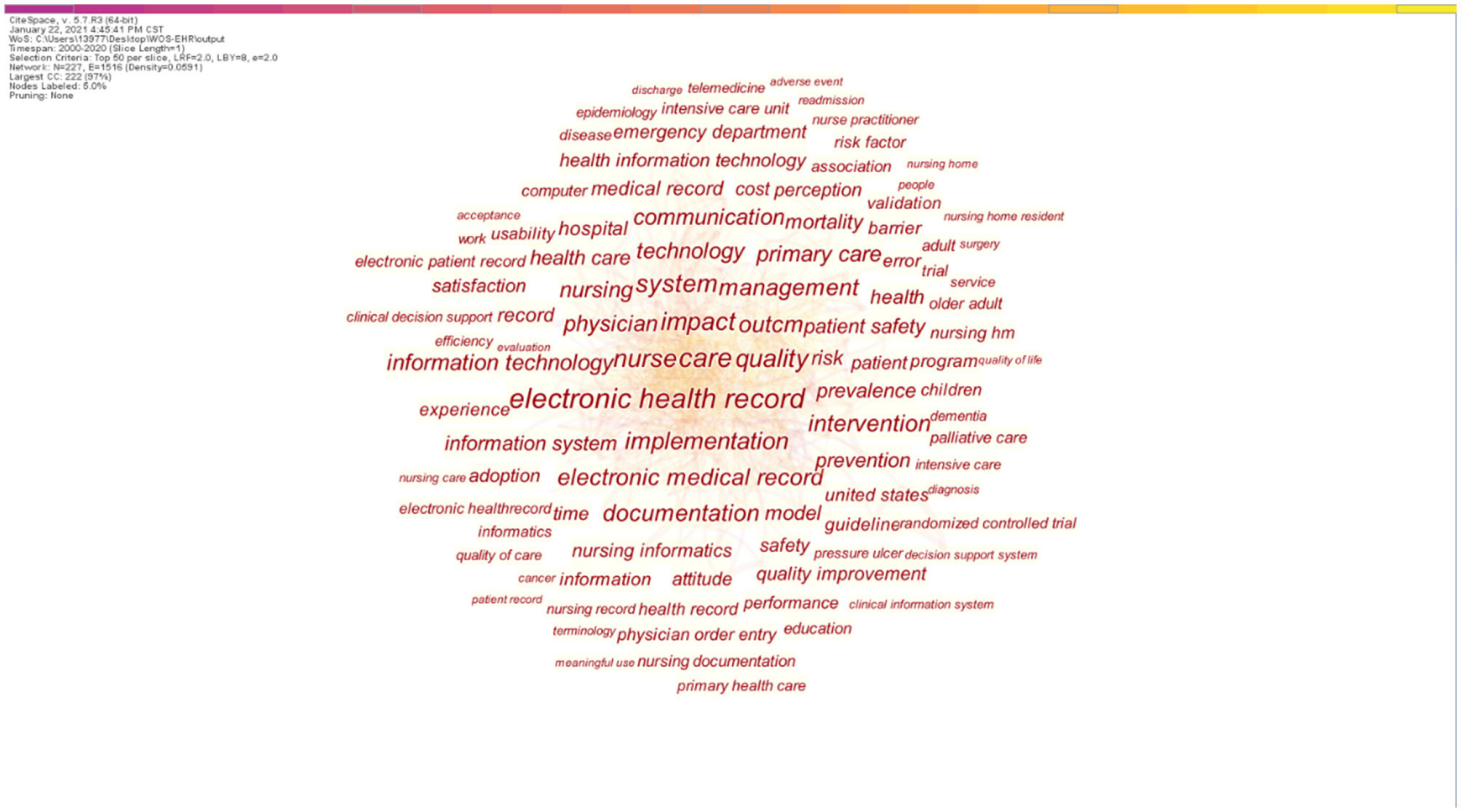
Research keywords visual analysis.

**Figure 8:**
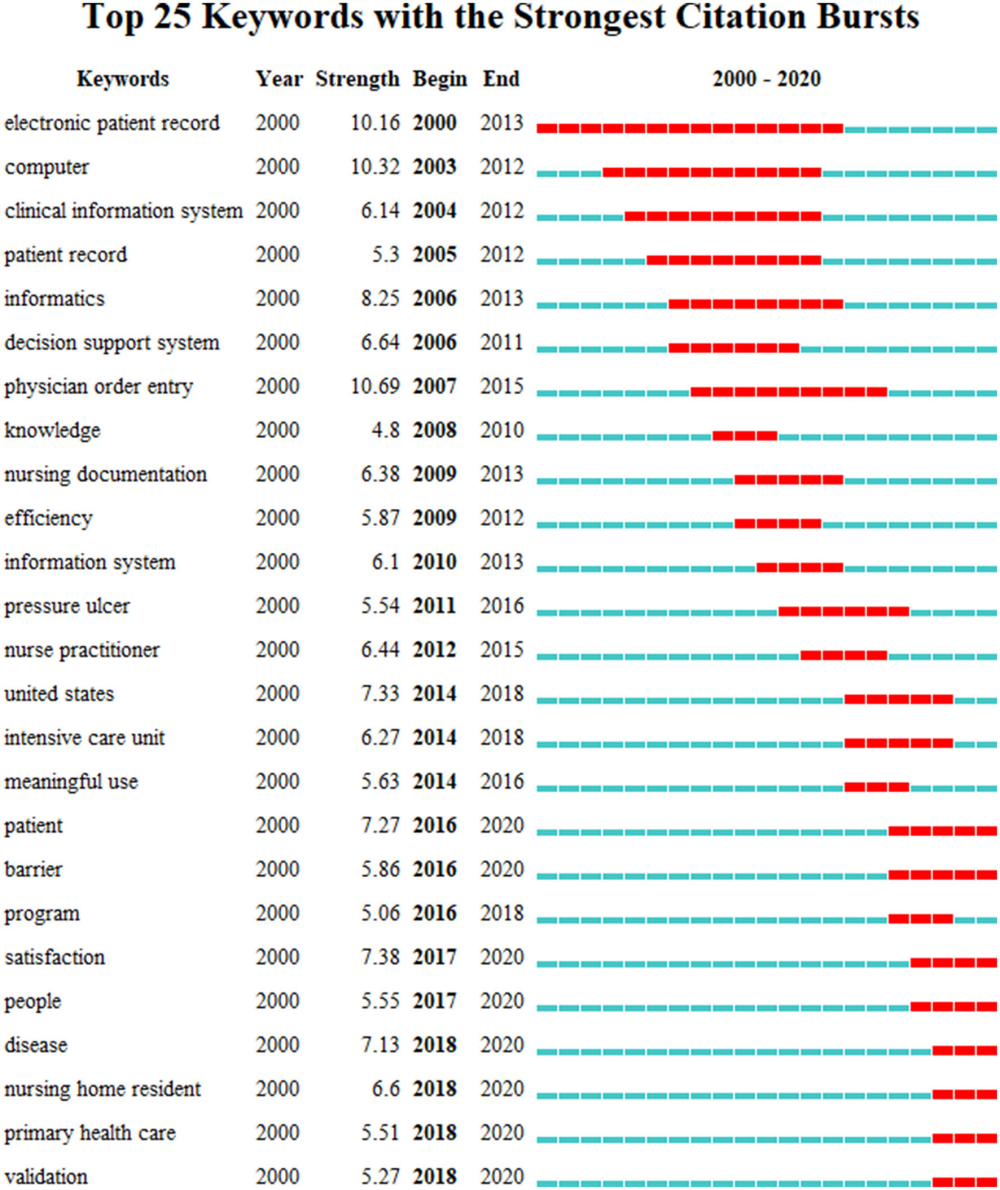
Keywords with the citation bursts of research from 2000 to 2020.

A total of 227 research keywords were identified, revealing the most-researched topics (Figure 7). Set the co-occurrence frequency threshold to filter out these keywords with low co-occurrence frequency for better analysis. The figure shows co-occurrence when thresholds were set to 20, including 74 keywords. From 2000 to 2020, there seem to be citation outbursts on 25 keywords, showing the largest research activity in the field of nursing EHR (Figure 8). The time interval is plotted on the blue line, and the period of the outbreak keyword is highlighted in red. Sort by the start time of the emergency.

### 3.6. References-based co-citation analysis

**Figure 9:**
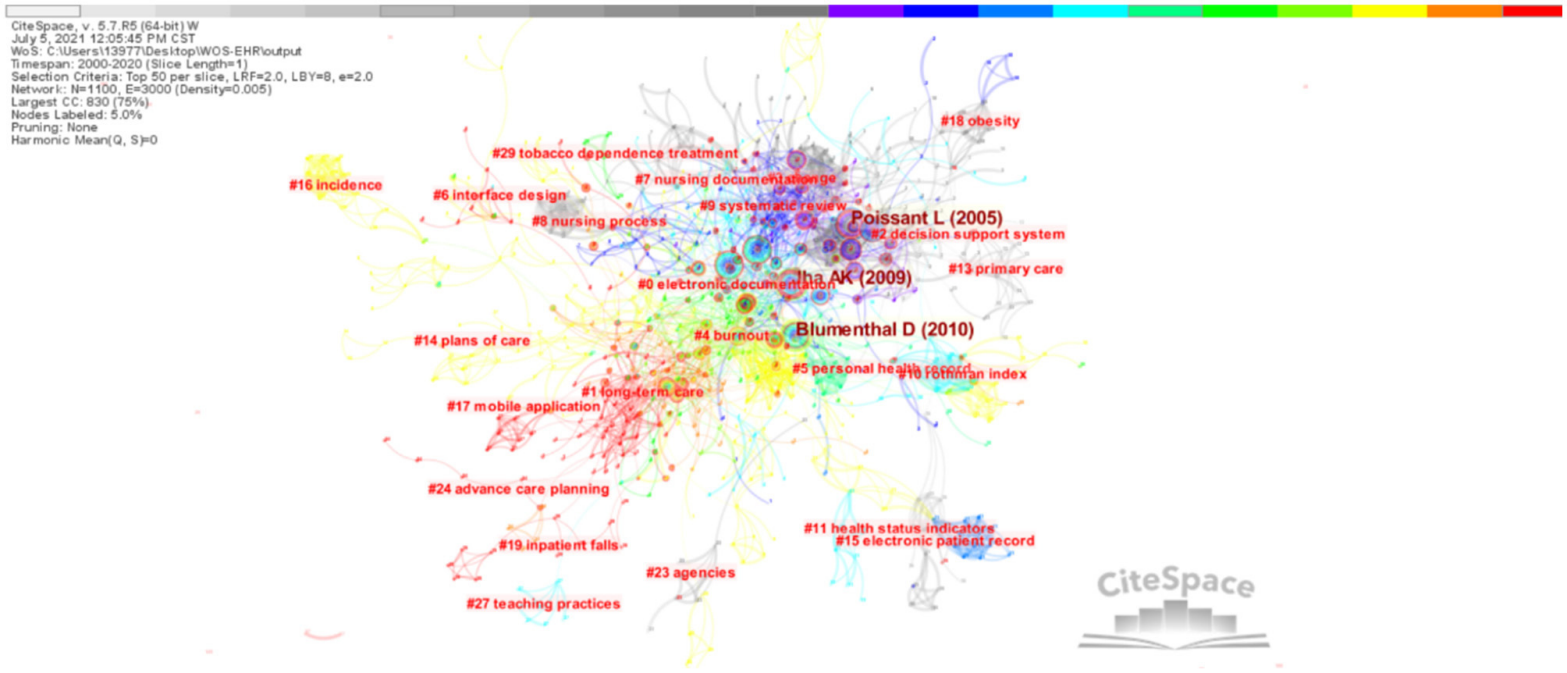
Cluster visual analysis of EHR research in the field of nursing.

**Figure 10:**
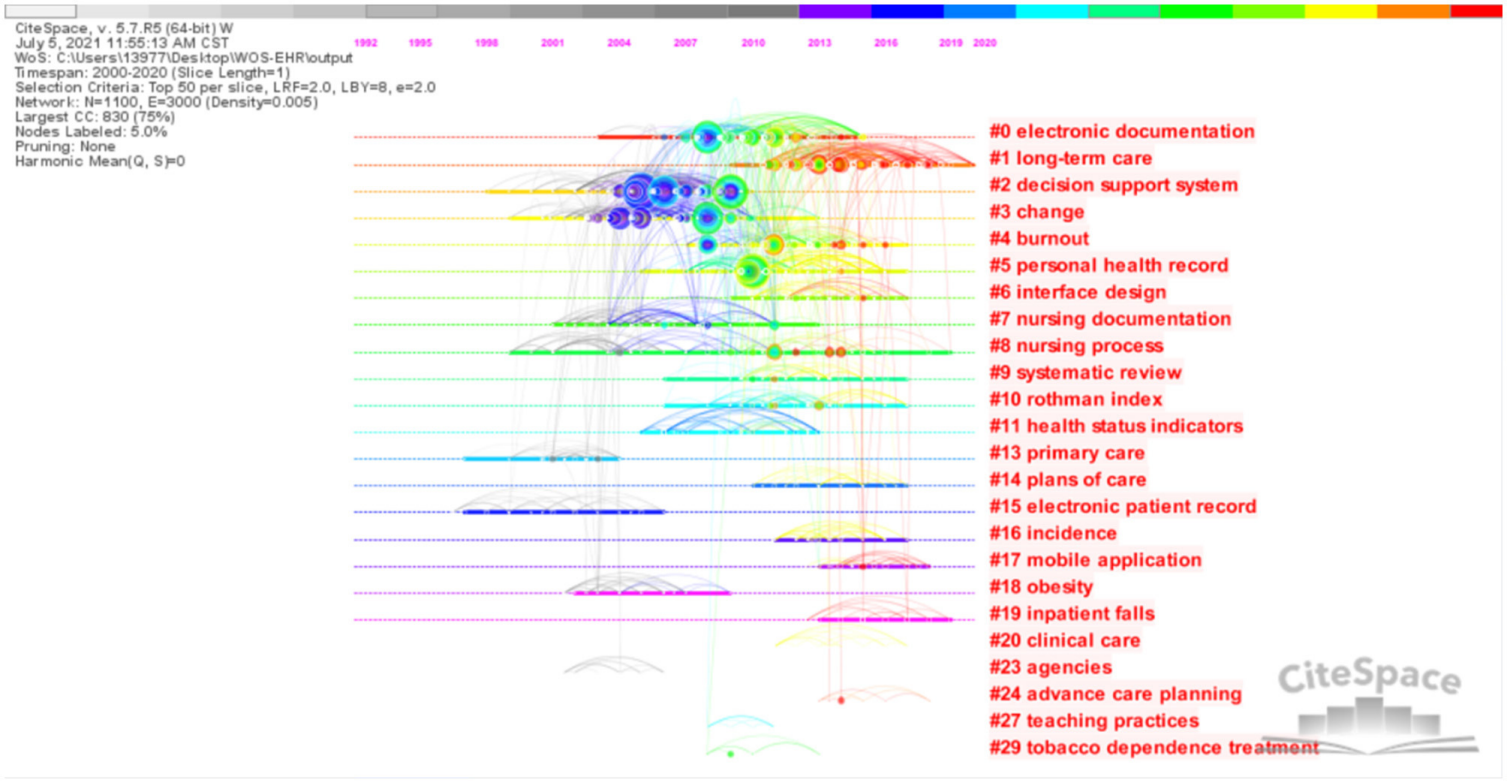
Cluster timeline visual analysis of EHR research in the field of nursing.

A total of 2616 qualified records were cited by 32,479 references. The network is divided into 24 co-citation clusters. The cluster analysis produced a simple clustering network (Figure 9) and timeline view (Figure 10), using a log-likelihood ratio (LLR) text mining method to name each cluster. It can be seen from the figure that Jha (17), Poissant (18), and Blumenthal (19), these three articles are very important because the high frequency of citations shows that they are of great significance in this field.

It can be seen from Figure 10 that the research fields are different in different periods. There are 4 clusters (#1 long-term care, #17 mobile application, #19 inpatient falls, and #24 advance care planning) that are the main research areas with most publications dated around 2015, shown in red. In descending order of size, the top four clusters are #0 electronic documentation, #1 long-term care, #2 decision support system, and #3 change. More detailed information about some clusters is presented as follows.

### 3.7. Top 10 leading highly cited articles

Highly cited articles can be used to identify the key knowledge base of each research field (20). The top 10 most cited publications are shown in Table 3 (18, 21–29). These articles mainly focus on the application of EHR in primary health care, as well as the views of medical staff and the impact on patients.

**Table 3:** Top 10 most cited articles in research.

**Rank**	**Citation counts**	**References**	**Time**	**Title**	**Abbreviations of journals**
1	891	Bakitas M et al. (21)	AUG 19 2009	Effects of a Palliative Care Intervention on Clinical Outcomes in Patients With Advanced Cancer The Project ENABLE II Randomized Controlled Trial	*JAMA*
2	579	Hayrinen K et al. (22)	MAY 2008	Definition, structure, content, use, and impacts of electronic health records: A review of the research literature	*Int J Med Inform*
3	479	Poissant L et al. (18)	SEP-OCT 2005	The impact of electronic health records on time efficiency of physicians and nurses: A systematic review	*J Am Med Inform Assn*
4	304	Clegg A et al. (23)	MAY 2016	Development and validation of an electronic frailty index using routine primary care electronic health record data	*Age Aging*
5	262	Bolton-Moore C et al. (24)	OCT 24 2007	Clinical outcomes and CD4 cell response in children receiving antiretroviral therapy at primary health care facilities in Zambia	*Jama*
6	231	Hobbs FDR et al. (25)	JUN 4 2016	The clinical workload in UK primary care: a retrospective analysis of 100 million consultations in England, 2007-14	*Lancet*
7	227	Stellefson M et al. (26)	FEB 2013	The Chronic Care Model and Diabetes Management in US Primary Care Settings: A Systematic Review	*Prev Chronic Dis*
8	202	Coiera EW et al. (27)	MAY 6 2002	Communication loads on clinical staff in the emergency department	*Med J Australia*
9	201	Pronovost P et al. (28)	DEC 2003	Medication reconciliation: A practical tool to reduce the risk of medication errors	*Crit Care*
10	161	Hewitt ME et al. (29)	JUN 1 2007	Perspectives on post-treatment cancer care: Qualitative research with survivors, nurses, and physicians	*J Clin Oncol*

## 4. Discussion

Academic work in this area has shifted from focusing on the popularization of EHR to the current stage of application. The efficient and safe use and development of EHR are also regarded as an important direction in the nursing field.

Our research shows that the number of EHR publications in nursing research has continued to grow in recent years. This trend may be inseparable from the widespread application of hospital information management. The use of EHR in health facilities is already a rule rather than an option (30), and nursing staff is also exploring knowledge using EHR. In addition to the wider use of EHR in hospitals, many articles explore the pros and cons of using EHR in other institutions such as nursing homes (31, 32).

Collaborative networks reveal collaborative links between institutions and countries. The adoption of the Health Information Technology Adoption Act in the United States in 2009 resulted in high penetration of EHR (33). Analysis of subject categories and journals can help understand research directions for a particular subject. There are also more and more articles published in nursing journals, indicating that EHR is also valued by nurses and will better address clinical nursing problems. The co-speech category shows that healthcare science and services, medical informatics, and computer science are the three most active subject categories. It can be seen that with the popularization of EHR, medical informatization and the combination of medicine and computing have become research hotspots. Brom et al. used machine learning methods to identify patients with potential readmission in a hospital in EHR for better allocation of care resources (34). Lin et al. applying a “Practical Informatics” Strategy During COVID-19 Using EHR to Meet Demands Created by Mass Influx into Health Systems (35). Lopez et al. also identified electronic health record completion as an outcome measure for pressure ulcers, falls, and social vulnerability risk during the COVID-19 epidemic, although this may also increase the physical and mental burden on health care workers during the pandemic (36, 37).

As can be seen from the keyword co-occurrence analysis and citation burst, EHR is in the stage of a continuous validation and overcoming obstacles. Cluster #1 “long-term care”: This cluster mainly discussed the problem faced by seniors and vulnerable populations that require 24-hour nursing care. Patients in need of long-term care have more and more opportunities to enter nursing homes, some of which are best described by HER (38, 39). During these transitions, communication must be carried out on multiple levels to ensure a smooth transition between the patient and the receiving organization, the smooth transfer of records between organizations, the maximization of results, the minimization of hospital stays, and where possible To reduce the possibility of rehospitalization (38).

Cluster #17 “mobile application”: The literature in this cluster describes that there has been a shift from implementing EHR to optimizing these systems, and the long-term issue of patient data operability is becoming more and more important. People are increasingly interested in obtaining medical information from hospital records and databases and providing portable records for convenient patient control (40).

Cluster #19 “inpatient falls”: This clustering literature covers adverse events, such as inpatient falls. This research topic has been a long-standing research hotspot. An inpatient fall is a preventable adverse event that can be managed more effectively and efficiently through a data-driven predictive approach (41). Nakatani et al. implemented a new approach and explored its effects in neurologic inpatient units. The results suggest that integrating an automatic fall prediction system with the EMR system could reduce inpatient falls (42).

Cluster #24 “advance care planning”: It mainly refers to the application of EHR. Advance care planning can ensure that care meets the wishes and needs of the patient. However, due to the failure to allow patients to participate in the planning in advance, the inability to obtain previous documents, or the poor quality of the documents, there is often a lack of advance care planning in actual clinical work. The standardized use of EHR helps to solve this problem. Kruse et al. used intervention tools in EHR to address these obstacles (38).

Difficulties remain in the application of EHR: EHR are not simply the transfer of paper content to an electronic information system. Some studies have compared the electronic health record and the paper version. There is not much difference in content, and the record format and terminology are not uniform, which weakens the original advantages (30, 43). In addition, may it take longer for nurses who are too old or new to the profession to learn how to use it, which also increases the workload of nurses in their daily work (44–46). What needs to be done in the future is to add plug-ins or tools to the electronic health record to assist the medical staff to understand the patient's condition, so that the electronic health record is not only a function of recording, but it is the most important to discover the function behind it and apply it to clinical work. It is also necessary to enhance the adaptability of nurses to EHR and improve computer-quality education to promote and innovate electronic health record systems that better meet the needs of clinical work (47).

## 5. Limitations

This study still has certain limitations. Firstly, a single database (WoSCC) was utilized. Future research can be extended to other databases. Secondly, since EHR does not have a unified definition in different countries and regions, there may be omissions in the retrieval process. Future research can increase the scope of EHR retrieval or hope that the standardized terminology of EHR will be unified. The search was limited to the English language. Therefore, a large number of articles in other languages may not be included. Finally, the peak period of cited references is 2–3 years, many studies have shown that EHR provides an effective guide for COVID-19 patients to diagnose problems, plan and implement appropriate interventions (48), or predict mortality and complication (49). However, due to the low frequency of citations that are not displayed in the visual view, there may also be similar problems other than COVID-19. In the future, this field should be explored at reasonable intervals.

## 6. Conclusion

Between 2000 and 2020, research related to EHR in the field of nursing increased year by year. Network map of countries and institutions showing strong collaboration between four countries and six institutions. The United States is the most influential country, University Penn in the United States is the most productive institution. The *Journal of American Medical Informatics Association* is the most cited. This study also reveals key aspects of EHR research and changes over time through co-occurrence, burst keyword analysis, and cluster analysis, but in the future, more research on EHR in the field of nursing is still needed.

## Data availability statement

The original contributions presented in the study are included in the article/supplementary material, further inquiries can be directed to the corresponding author.

## Author contributions

Data were collected and analyzed by ZL, YG, SD, and NW. ZZ and XP designed the study and drafted the manuscript. ZZ and YC obtained article information. ZL, NW, and YC revised the manuscript. All authors contributed to the article and approved the submitted version.
